# Methods of Population Spatialization Based on the Classification Information of Buildings from China’s First National Geoinformation Survey in Urban Area: A Case Study of Wuchang District, Wuhan City, China

**DOI:** 10.3390/s18082558

**Published:** 2018-08-04

**Authors:** Linze Li, Jiansong Li, Zilong Jiang, Lingli Zhao, Pengcheng Zhao

**Affiliations:** 1 School of Remote Sensing and Information Engineering, Wuhan University, Wuhan 430072, China; 2011301610391@whu.edu.cn (L.L.); Jiansongli@whu.edu.cn (J.L.); zhaolingli@whu.edu.cn (L.Z.); pengcheng.zhao@whu.edu.cn (P.Z.); 2 State Key Laboratory of Information Engineering in Surveying, Mapping & Remote Sensing, Wuhan University, Wuhan 430072, China

**Keywords:** population spatialization, multi-level method, China’s first national geoinformation survey, correlation analysis, overlay analysis

## Abstract

Most of the currently mature methods that are used globally for population spatialization are researched on a single level, and are dependent on the spatial relationship between population and land covers (city, road, water area, etc.), resulting in difficulties in data acquisition and an inability to identify precise features on the different levels. This paper proposes a multi-level population spatialization method on the different administrative levels with the support of China’s first national geoinformation survey, and then considers several approaches to verify the results of the multi-level method. This paper aims to establish a multi-level population spatialization method that is suitable for the administrative division of districts and streets. It is assumed that the same residential house has the same population density on the district level. Based on this assumption, the least squares regression model is used to obtain the optimized prediction model and accurate population space prediction results by dynamically segmenting and aggregating house categories.In addition, it is assumed that the distribution of the population is relatively regular in communities that are spatially close to each other, and that the population densities on the street level are similar, so the average population density is assessed by optimizing the community and surrounding residential houses on the street level. Finally, the scientificalness and rationality of the proposed method is proved by spatial autocorrelation analysis, overlay analysis, cross-validation analysis and accuracy assessment methods.

## 1. Introduction

Population data are one of the most direct indicators of human activity [1]. With the development of China’s urbanization process from 1949 to 2015, the proportion of the urban population in China increased from approximately 10% to 57.35% [2]. The spatial distribution of the population, population flow, and population structure are becoming increasingly important for the development of cities. The spatial distribution of the population influences not only the urbanization process and living environment [3,4], but also the development plan of the regional public education system, medical facilities, and other services, which are related to people’s vital interests [5,6,7].

The spatial distribution of the population is affected by many factors, such as geographic location, land cover, convenience of road networks, water areas, and economic development [8,9]. Therefore, traditional research methods mainly fit spatial population distributions by studying the coupling relationship between regional population density and its influence factors. Liao Shunbao et al. [10] examined the correlation between the population density and land use in Tibet and Qinghai Province and proposed a spatial model of population through multi-source data fusion method. Du Guoming et al. [11] used the data from the fifth census of Shenyang City and residential areas data in order to simulate population distribution through the spatial interpolation method. Given the shortcomings of the current spatial methods for urban populations, Kang Tingjun et al. [12] developed a multi-agent-based urban population distribution method. Using North Korea’s district-level census data, Shi Tingting et al. [13] analyzed the relationship between North Korea’s population density and spatial factors, and then performed multiple regression analysis to spatial status of North Korea’s population density. Dong Chun et al. [14] combined population statistics data with geographical data and economic data to establish apopulation spatialization method, which examines the coupling relationship between population distribution and related factors in a certain region.

Remote sensing imagery provides a new idea for population spatial modeling [15,16]. Many scholars use the advantages of remote sensing imagery, including its multilevel nature and high degree of timeliness, combined with geographic information system (GIS) technology to buildpopulation spatialization model at different spatial levels [17,18,19,20]. Chen Qing et al. [21] studied the correlation between night-time remote sensing images and geographic factors, performing a population spatialization experiment in the highly efficient eco-economic region in the Yellow River Delta. Lo C P et al. [22] studied the relationship between the gray value of thematic mapper (TM) images in different bands and urban population density. Li Shujuan et al. [23] used high-resolution remote sensing image to extract building information for different functions and calculated the population accommodation coefficients of different buildings in order to establish the spatial distribution map of the urban population. Wang Shixin et al. [24] used three-dimensional (3D) reconstruction technology to identify and extract urban residents’ housing areas and elevation in ZY-3 images.

Typically, in population census data, the statistical unit is the administrative unit, thus, the statistical level is relatively coarse and the types of data are limited. Therefore, the selection and improvement of mathematical methods are crucial for obtaining high-precision population spatialization results. Commonly used methods include geostatistics methods [25], spatial regression models [26,27], spatial interpolation methods [28,29], and machine learning methods [30,31]. Holt et al. [32] used the improved population weight method to interpolate census data spatially, and this method can better explain the spatial distribution of the population within the census administrative division. Wang Keijing et al. [33] studied population spatialization by using multivariate statistical regression and geo-weighted regression (GWR) models. Cao Li-qin et al. [34] predicted the population of 76 districts or counties in Hubei Province in 2002 by using the neural network model to establish a relationship between the brightness of nighttime light data and the urban population.

The study of population spatialization has become more comprehensive given the integration of more data sources [35,36] and technological methods [37,38]. At present, there are a number of mature data sets of population spatialization achievements covering the world, countries, or regions, such as Landscan [39], Worldpop [40], and GHS-POP [41]. These data sets provide detailed and accurate results of population maps of dynamic population flow [42,43,44], age structure change [45,46,47], urbanization development [48,49,50], building or settlement characteristic information [51,52,53], and greatly promote the cross-study of population spatialization. By combining with other related fields, important data and method support are provided to guide the urban planning [42,54], to assess the risk of demographic risk [55,56], and to improve the population quality of life [57,58].

Facing thefact that the fusion of more and more data sources, the variety method of population spatialization and the difference perspective of population research, it is a very important direction of the future research to use suitable data and establish the population spatialization method to meet the needs of different administrative units. At present, many scholars have carried out a series of researches on data process and methods for the improvement of the data source precision [54], the cross validation of population spatialization method [45,47], and the evaluation of the experimental results [31]. Few people pay attention to the demand and difference of the population spatialization method under different administrative units. Based on this, this paper makes use of data sources to establish population spatialization methods under the perspective of different administrative units, and tries to establish a reasonable method system of population accuracy evaluation to verify the rationality of the experimental results.

The China’s first national geoinformation survey started in January 2013 and lasted three years. Its purpose was to systematically obtain the authoritative, objective, and accurate information on the geographic conditions of the country in order to provide an important data foundation for promoting ecological environmental protection and building a resource conserving and environmentally friendly society. Through synthetically using the global navigation satellite system (GNSS), aerospace remote sensing (RS) technology, GIS technology, and other modern surveying and mapping technology, the survey can dynamically and quantitatively recognize land surface morphology, land covers, build-up zones, and monitor the spatial distribution and development of resources, the environment, ecology, and economic factors. This data set mainly contains three types of data: land topography data (DEM, Slope data), land cover classification data (“LCA”, which contain 10 major categories, such as farmland, garden land, woodland, and more than 100 smaller categories), and social geographical units, including point of interests (“POIs ”, educational facilities, hospitals, and so on), administrative unit categories and vector boundaries and other urban integrated functional units (“BUCA”, “BUCP”). The greatest advantage of this data set is that it is highly accurate in building space information, such as building location, shape, and other characteristics, including building types and height. Such detailed information on building classification provides useful data for the study of population spatializationbased on the housing construction area.

The main innovations of this paper are as follows: (1) The spatial and attribute information of buildings in China’s first national geoinformation survey is fully mined. Through the combination of different administrative divisions and thresholds for the proportion of housing construction areas, this paper gives functional attributes to all buildings and screens out residential houses. (2) A multi-level population spatialization method that is applicable to different administrative unit levels is established. (3) Various methods are used to qualitatively and quantitatively study differences in the experimental results on thedifferent levels. Thecommon and differences are well analyzed and explained. 

## 2. Data Acquisition and Preprocessing

### 2.1. China’s Administrative Classification

The current administrative divisions in China are as follows: The first level is the provincial administrative units, which mainly include provinces, autonomous regions, municipalities, and special administrative regions. The second level is the prefectural administrative regions, which are divided into prefecture-level cities and regions. The third level is the county-level administrative units, which mainly include municipal districts, counties, and county-level cities. The fourth level is the township-level administrative units, which mainly include streets and townships. The fifth level is the village-level administrative units, which mainly include communities and administrative villages. The latter is a group-level administrative village that is divided into natural village groups and community residential groups [59,60]. The research objects in this paper are mainly the street and community units under the municipal district, corresponding to the fourth and fifth levels of the administrative division. Residential quarters, however, are not administrative units; rather, contains residential houses and are equipped with commercial outlets, culture and education, entertainment, and other public facilities [61].

### 2.2. Data Acquisition

The specific data needed for the multi-level population spatialization method are shown in Table 1: (1) Spatial vector data, including street administrative units, community administrative units, and housing construction features, are mainly collected from China’s first national geoinformation survey. (2) Demographic data, including resident population in sub-districts and resident population in sub-communities, are mainly collected from the Wuhan Statistical Yearbook in 2015, The Sixth National Population Census, and the Wuhan Community Demographic Census in 2013. 

### 2.3. Data Preprocessing

**Figure 1:**
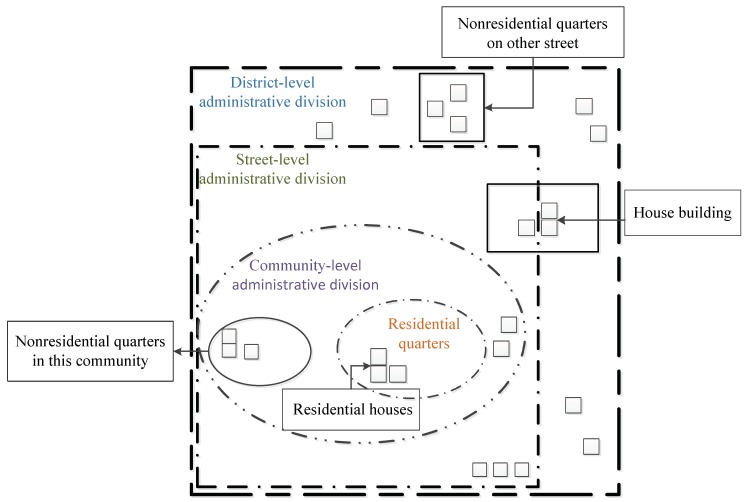
Schematic Diagram of Data Preprocessing.

Due to differences in the administrative vector boundaries, as collected by China’s first national geoinformation survey and the Community Demographic Census, the administrative boundaries corresponding to house buildings are not completely consistent. At the same time, as some community administrative boundaries are adjusted or merged with other communities, a small part of the “community vacancy” area may exist in the data results, that is, there are no community attributes for the house buildings in this area. In addition, because of the error in the boundaries of the residential quarters, some house buildings do not have attributes of residential communities. Therefore, data preprocessing mainly corrects the boundaries of administrative units, and defines the three types of attributes, including street, community, and residential quarters, clearly corresponding to the house buildings. The schematic diagram of data preprocessing is showed in Figure 1.

(1) Take the vector boundary of non-residential quarters from the BUCA layer and the BUCP layer as the standard. Then, review all of the house buildings in the LCA layer and delete those belonging to non-residential quarters by using spatial location query and attribute query functions.
(2) Take the vector boundary of residential quarters from the BUCA and BUCP layer as the standard to merge the house buildings in the LCA layer. Then, add the corresponding residential quarters attribute information to these house buildings through the spatial location query and attribute query functions.
(3) Review the remaining house buildings in the LCA layer. Then, take the community vector boundary as the standard to merge house buildings belonging to the same community and add the corresponding community attribute information through the spatial location query and attribute query functions.
(4) Use the street boundary to determine the street information for all communities through the spatial location query and attribute query functions. Then, add the corresponding street attribute information that is not available at the community level and residential quarters.

## 3. Multi-Level Method and Experimental Verification

**Figure 2:**
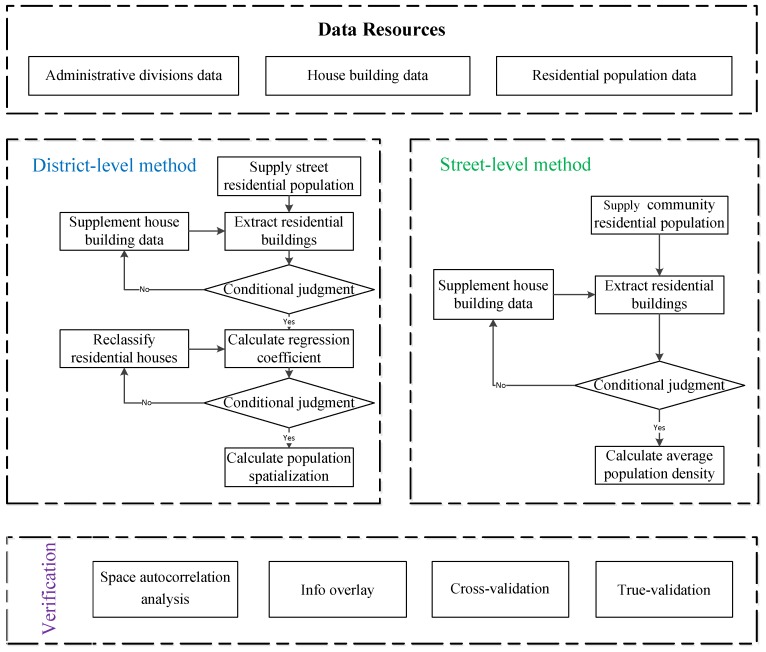
Workflow of Multi-level Population Spatialization Method and Verification.

Following data preprocessing, the multi-level population spatialization method is established for large-level regions, such as the district level and small-level areas, such as the street level. Then, space autocorrelation analysis, overlay analysis, and cross-validation analysis are used to verify the rationality of these methods. The workflow of multi-level population spatialization method and verification is showed in Figure 2.

### 3.1. The Method on the District Level

The main basis for the population spatialization method on the district level is as follows: sub-streets in China are mostly located in the urban centers or urban development areas; the layout of this region’s residential houses is more uniform than that in other areas. According to the standard of China’s first national geoinformation survey, residential quarters that are of the same type have high degree of similarity in terms of floor height and construction area. Therefore, the population density of different types of residential houses should be estimated by least squares regression through the classification that is based on the assumption that residential houses of the same type have the same population density [62].

**Figure 3:**
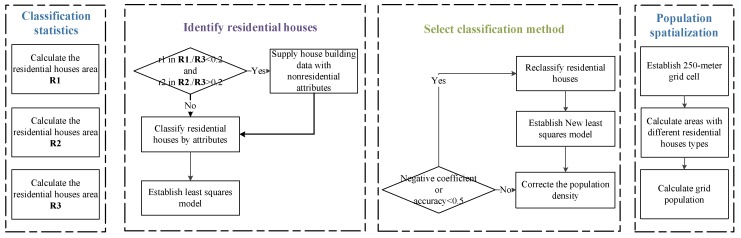
Workflow of the Population Spatialization Method on the District Level.

As showed in Figure 3, the first step in processing the data is to calculate the areas of residential houses with attributes of the residential quarter, and then count the total areas of residential houses on each street and record them in vector ***R1***. The second step is to calculate the house building areas that do not have community attributes but have street attributes, and then count the total areas of house buildings on each street and record them in vector ***R2***. The next step is to count all of the house building areas with street attributes, and then count the total areas of house buildings on each street and record them in vector ***R3***. The residential population of each street is evaluated by using the Wuhan Statistical Yearbook and The Sixth National Population Census.

Field surveys and expert verification indicate that residential houses are not extracted fully if the total house building area with residential quarter properties is less than 20% of the total street house building area and if the house building area with non-community properties exceeds 20% of the total street house building area. Thus, the ratios ***R1/R3*** and ***R2/R3*** must be calculated. If the results of the ratios meet the above conditions, then all of the residential houses have been extracted and residential house areas have been stored in ***R1***. Otherwise, the house buildings with community attributes and non-residential quarter attributes should be regarded as residential houses and addedin***R1***. Then, the residential houses should be classified into multi-floor buildings, multi-floor independent buildings, low-floor buildings, and low-floor independent buildings (the detailed description of house buildings is showed in Table 2), according to the attributes and enter the final residential house areas into Equation (1).

(1) $$y_{i}=a_{1}S_{1i}+a_{2}S_{2i}+a_{3}S_{3i}+a_{4}S_{4i}+\Delta_{i}\;(i=1\ldots n)$$

where $y_{i}$ represents the resident population count for each street, $a_{1}-a_{4}$ represent different housing density coefficients, and $S_{1i}-S_{4i}$ represent the areas of the different type of residential houses. $\Delta_{i}$ represents random error term under the assumption of normal distribution and n represents the number of streets.

First, the coefficients and goodness of fit of the results must be evaluated. If some of the coefficients are negative or the overall goodness of fit is less than 0.5, multi-floor independent houses and multi-floor houses should be combined into a new type of house building, and low-floor independent houses and low-rise houses should be merged into another new type of house building. Then, the residential houses should be reclassified. The improved model is shown in Equation (2):

(2) $$y_{i}=a_{1}(S_{1i}+S_{2i})+a_{2}(S_{3i}+S_{4i})+\Delta_{i}\;(i=1\ldots n)$$

The fitted population can be calculated through the coefficient results. However, because there is a certain deviation between the fitted population and the actual population count, the coefficients must be corrected [14,38] by using Equation (3).

(3) $$a_{i}^{\prime }={\left({\vec{y}}_{i}/y_{i}\right)}^{-1}\times a_{i}\;(i=1,2)\;$$

Finally, it is appropriate to establish 250-m spatial grid cell, and the corrected coefficients are used to estimate the population count in each grid cell based on the areas of the different residential houses.

### 3.2. The Method on the Street Level

As the second smallest administrative unit in China, the community covers between a few to more than ten residential quarters, and the community areas are much smaller than the street areas. Therefore, the residential house areas of the community are far smaller than the street areas. The least squares method is suitable when the sample size is large and the variation in the independent variable is small, the resulting error of this method may be too large in order to fit the estimated community population. The population distribution is relatively regular in a community with close spatial distances, and the type of residential houses is uniform; thus, residential houses that are in close distances to each other have similar population densities. In this paper, it is reasonable to calculate the average population density of residential houses on the community level [37,63]. 

**Figure 4:**
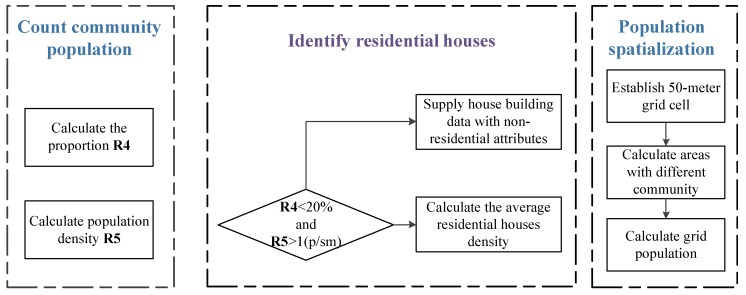
Workflow of the Population Spatialization Method on the Street Level.

Using the first step of the Figure 3, the total area of residential houses in each residential quarter should be counted and recorded in vector ***C1.*** Then, the total area of house buildings in each community should be counted and recorded in vector ***C2.*** The residential population of each community from the *Wuhan Community Demographic Census* is recorded in vector ***P***. The workflow of the population spatialization method on the street level is showed in Figure 4. 

Field surveys and expert verification indicate that residential houses are not fully extracted if the total house building area with residential quarter properties is less than 20% of the house building area with non-residential quarter properties, and if the average population density of residential houses is above one person per square meter. Thus, the ratios ***R4*** = ***C1/C2*** and ***R5**** = **P******/C1*** must be calculated. If the results of the ratios meet the above conditions, then all of the residential houses have been extracted and residential house areas have been stored in ***C1***. Otherwise, the house buildings that have community attributes but do not have residential quarter attributes should be regarded as residential houses, and the area should be added the area into ***C1***.Finally, the final results of the residential house areas should be entered into Equation (4).

(4) $$y_{i}=P_{i}/C1_{i}\;(i=1,\ldots n)$$

Finally, it is appropriate to establish 50-m spatial grid cell, count the areas of residential houses in each grid cell and calculate the total population.

### 3.3. Experimental Result Verification

The results of different administrative units are obtained by using the multi-level population space model, but whether the results of the experiments are reasonable requires further verification. Therefore, in this paper, three qualitative and quantitative analysis methods are utilized. Detailed procedures are described in Section 3.3.1, Section 3.3.2 and Section 3.3.3.

#### 3.3.1. Spatial Autocorrelation Analysis

Spatial autocorrelation refers to the potential interdependence of some variables within observation data in the same distribution area. The local Moran index (Lisa index) [64,65] is a classical algorithm that is used to detect local spatial autocorrelation. It can reflect the spatial aggregation condition well by calculating the index of the correlation between the spatial unit and the neighborhood. At the same time, this method can take the variability of the local state into account when calculating the global index. Therefore, in this paper, this method is used to explore the spatial autocorrelation of the population. 

#### 3.3.2. Spatial Overlay Analysis

Overlay analysis creates new feature layers by stacking two-layer or multilayer map elements, not only generating new spatial relationships between multiple features, but also linking their attributes [14,26]. Geographic locations (governments), educational resources (primary and secondary schools), medical and health resources (hospitals on the different levels, community service centers), and road networks (traffic trunks) are selected in this paper. By calculating the average shortest distance and coverage degree, this paper analyzes the dominant resource advantage and influence degree in the population agglomeration areas, demonstrating the rationality of the population spatialization results. 

The average shortest distance refers to the hierarchical statistics of population spatialization results, and it is calculated as the average distance between the different population levels to the nearest government, educational resources, medical and health resources, and road network. Quantifying the linear relationship between different population levels and impact factors can reflect the rationality of the method, at the same time, can provide a deeper understanding of the impact of different factors on population distribution. 

**Figure 5:**
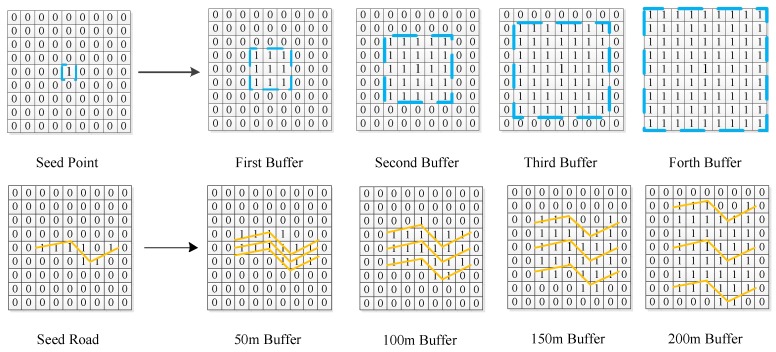
Multilayer Buffer Schematic.

Regarding these features as the center, a multi-layered buffer zone was established (the multilayer buffer schematic is showed in Figure 5). The service capabilities of the impact factors were explored through the statistics of the population covered by different buffer zones. For the point-like features, such as governments, educational resources, and medical and health resources, the domain expansion search is carried out by the topological relation by using the grid cell of the point-like feature as the center, and the total population in the buffer zone is counted. 

For the road network feature, buffers of 50 m, 100 m, 150 m, and 200 m are established on the district level, and the total population in the buffer zone is counted. To solve the problem of grid size mismatch between the two levels, this paper spatially aggregates the population results on the street level. The size of the merged grid is consistent with the population grid on the district level.

#### 3.3.3. Cross-Validation Analysis

Cross-validation is a model validation technique that is used to assess how the results of a statistical analysis will generalize to an independent data set [47,66]. In this paper, the two levels of the population spatialization results are used to analyze the rationality of the multi-level method and to explain the difference. The cross-validation formula is shown in Equation (5):

(5) $$Relative\;error=2\times (CS_{i}-SS_{i})/(CS_{i}+SS_{i})\;(i=1,2\ldots .n)$$

where $CS_{i}$ is the population of each grid cell on the district level. $SS_{i}$ is the population of each grid cell on the street level. n is the number of grid cells. To solve the problem of grid size mismatch between the two results, this paper spatially aggregates the population grid cells on the street level, such that it is consistent with the population grid cell on the district level.

## 4. Results and Discussion

Wuchang District is one of the areas in downtown of Wuhan City and it is adjacent to the Yangtze River and the Han River. This district is the political center of Hubei Province and is also the place where universities and talents converge. The total area is 107.76 km^2^, and the center is 30°33’56”in the north latitude and 114°18’90” in the east. The urbanization rate of Wuchang District reached 96.2% in 2010, and the district has consistently maintained an urbanization rate of 100% in recent years. The population in Wuchang District has maintained a growth rate of approximately 1% since 2010, implying that the population has steadily increased. In 2015, there are 14 street-level governments, 196 educational resources, 105 medical and health facilities, and a road network length of 338.84 km.

**Figure 6:**
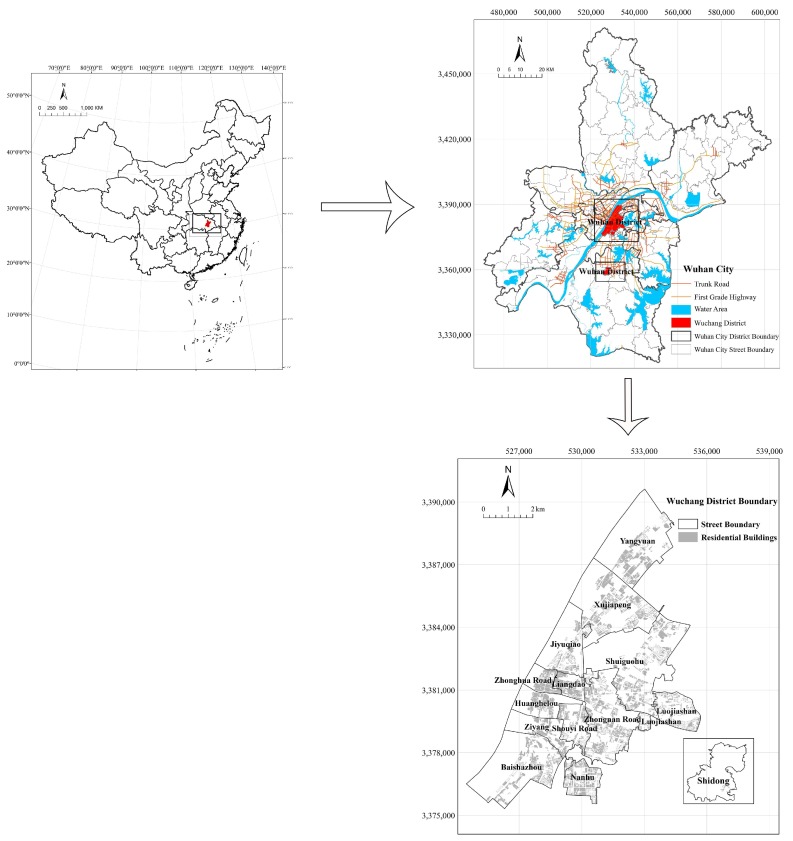
The Geographic Location of Wuchang District in Wuhan, China.

According to the administrative divisions, Wuchang District consists of the following 14 streets Baishazhou, Huanghelou, Jiyuqiao, Liangdao, Luojiashan, Nanhu, Shouyi Road, Shuiguohu, Xujiapeng, Yangyuan, Zhonghua Road, Zhongnan Road, Ziyang, and Shidong. The geographic location of Wuchang District is shown in Figure 6.

Wuchang District is selected as an experimental area for the following reasons:

(1) Wuchang District is located in the central urban area of Wuhan City, where the buildings are more concentrated and the types of buildings are more complicated. Therefore, the method will be scientific and universal if it has highly accurate results.
(2) Wuchang District contains 14 streets and 195 communities, and the data and information on house buildings are adequate.
(3) Wuhan City has conducted the *Community Demographic Census* since 2013. The granularity of statistical units is small, the population data sources are adequate, and the recency of the data is sufficient.
(4) Wuchang District has made many efforts to rebuild house buildings in recent years. If we can extract the spatial distribution of residential houses accurately, and remove abandoned buildings and other types of buildings to calculate the population spatialization results accurately, the study can provide important reference values for other cities with rapid urbanization.

### 4.1. The Results of the Population Spatialization Method on the District Level

**Figure 7:**
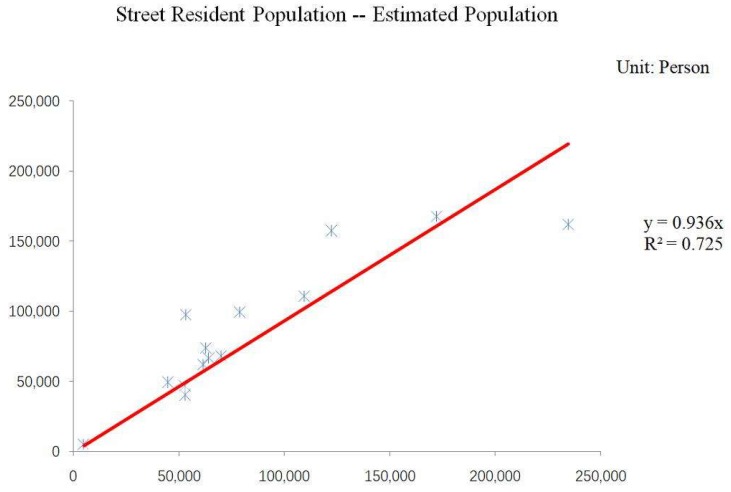
The Fit of the Street Population and Estimated Population.

In the experiment, it is not satisfactory to divide residential houses into four types because the results do not meet a conditional judgment. Thus, it is helpful to reclassify the residential houses. The correlation between the street population and estimated population when using the least squares regression model is shown in Figure 7.

The Figure 7 shows a clear linear correlation between the resident population and estimated population. The fitting coefficient reaches 0.936 and the goodness of fit is 0.725, which not only satisfies the conditional judgment, but also verifies that the experimental results have good accuracy.

According to the relative proportion between the estimated population and street population, it is reasonable to use Equation (3) to correct the population count of each type of house building. The corrected coefficient results are shown in Table 3.

As shown in Table 3, the relative proportion of almost all streets is basically less than 30%, except for Nanhu. The field investigation found that the main reason for this result is that the construction of Nanhu has been developing rapidly in recent years; the areas of house buildings have been increasing significantly, while the resident occupancy rate remains relatively low. The average fitting error in the Wuchang District is only 13.03%, indicating that the use of this method on the district level is reasonable. 

**Figure 8:**
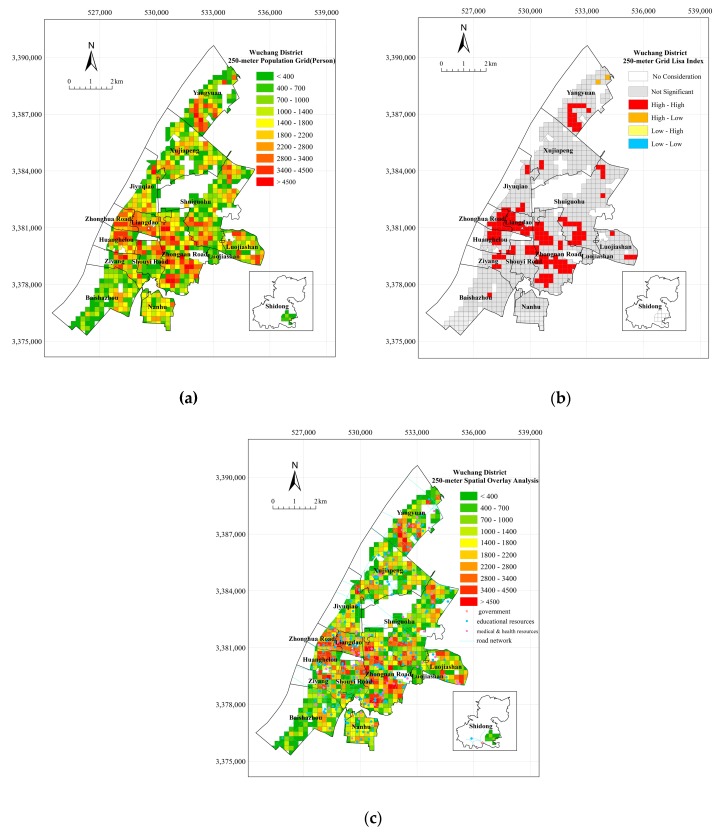
The Experimental Results and Analysis on the District Level ((**a**) Wuchang 250-m Population Grid; (**b**) The Result of Local Moran’s I; and, (**c**) Spatial Overlay Analysis).

According to the calculations of the population spatialization method on the district level, the number of population grid cells in Wuchang District is 1300 and the total population is approximately 1.21 million. The overall accuracy of the experiment reaches 99.98%, as the actual resident population is 1.182 million. The 250-m spatial population result for Wuchang District is shown in Figure 8.

As shown in Figure 8a, the areas with a large population are mainly distributed in the northern and middle-central areas of Wuchang District, including Yangyuan, Zhongnan Road, Zhonghua Road, Huanghelou, and Liangdao. The local correlation analysis result of the population spatialization is shown in Figure 8b. Most areas of the Wuchang District do not have an obvious spatial correlation of the population, especially Xujiapeng, Jiyuqiao, Luojiashan, Baishazhou, and Nanhu, where the population is small and the residents are scattered. In regions where the correlation is obvious, the vast majority of regions satisfy the “High-High” condition, and they are concentrated in Yangyuan, Shuiguohu, Zhonghua Road, Huanghelou, Liangdao, and Zhongnan Road. The residential houses in these areas are relatively compact, and large-level residential quarters have a spatial structure of “adjacent”. Therefore, these areas mainly provide living space that meets the needs of residents in terms of regional planning. 

**Figure 9:**
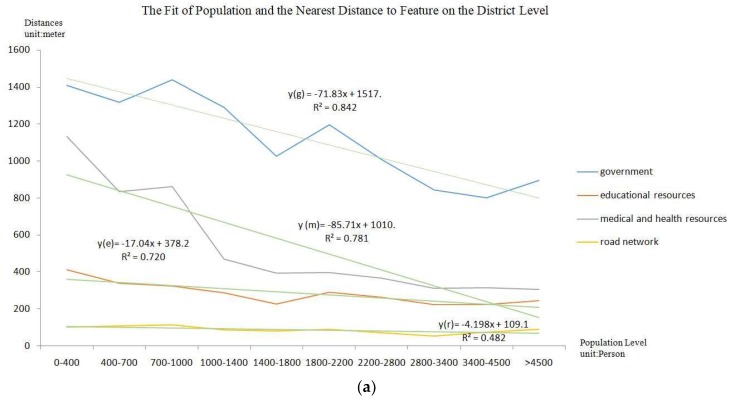

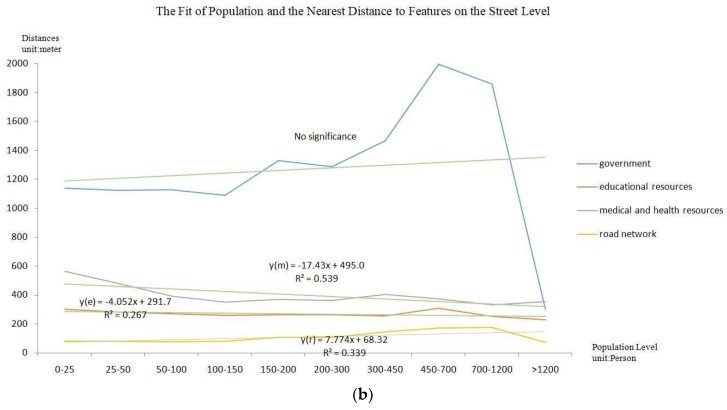
The Fit of Population and the Average Nearest Distance to Features ((**a**) result on the district level; and, (**b**) result on the street level).

As shown in Figure 8c and Figure 9 and Table 4, as the population increases, these factors obviously reduce the service distance, and the experimental results have better fit accuracy. Medical and health resources are most sensitive to population concentration, and, when compared with other features, these services are associated with a higher percentage of the population in the nearest buffers. With the expansion of urbanization, the areas surrounding the government are mainly replaced by commercial land, and the population coverage in different buffer areas shows that the population tends to move outward from the center of the street, but the location of governments remains highly attractive. The reasonable distribution and perfect construction of educational resources and road networks have a lesser influence on the population level. As shown by the overlay, the central region with a larger population has obvious advantages in terms of location and the strong construction of public service facilities. In the southern parts of Zhongnan Road and Shuiguohu, areas that are located on the main road of the Wuchang District, the road network is well developed and traffic is convenient. Although these areas are far from the government, they are also highly attractive to the population, leading to the development of education and medical facilities.

### 4.2. The Results of the Population Spatialization Method on the Street Level

**Figure 10:**
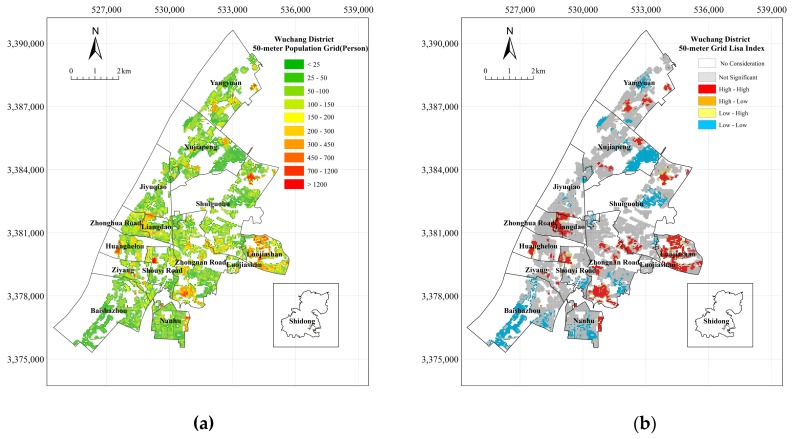

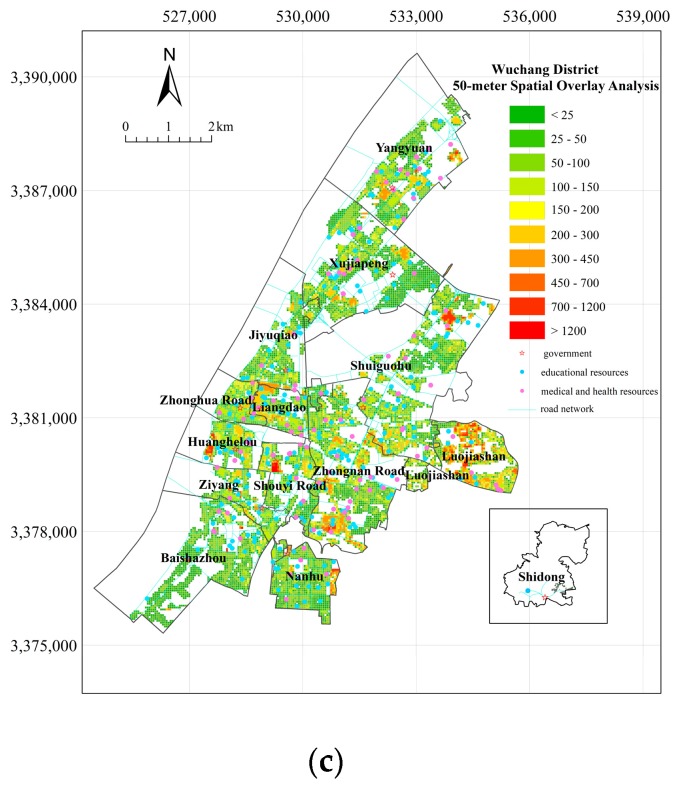
The Experimental Results and Analysis on the Street Level ((**a**) Wuchang 50-m Population Grid; (**b**) The Result of Local Moran’s I; and, (**c**) Spatial Overlay Analysis).

According to the calculations of the population spatialization method on the street level, the number of population grid cells in Wuchang District is 28,599 and the total population is approximately 1.22 million. The overall accuracy of the experiment reached 99.97%, as the actual resident population is 1.182 million. The experimental results are shown in Figure 10.

As shown in Figure 10a, the areas with large populations in Wuchang District are mainly distributed in the central region, including parts of Luojiashan, Zhongnan Road, Huanghelou, Shouyi Road, and Shuiguohu. In contrast to the results that are presented in Section 3.1, these results show that the development of road networks in some parts of Luojiashan and Shuiguohu is relatively common, but the population is also relatively large. This result is mainly due to the proximity of colleges and universities to these areas, as educational and medical resources are abundant. Areas with a small population are also more concentrated, and are mainly distributed in Xujiapeng, Baishazhou, and Nanhu. Educational and medical and health resources in these areas are relatively scarce.

Similarly, the local correlation analysis on the results of the population spatialization is shown in Figure 10b. The figure shows no obvious aggregation in most areas of Wuchang District. In addition, the “High-High” condition and “Low-Low” condition regions reflect the strong correlation of the population distribution in some areas of Wuchang District. The differentiation between high-aggregation regions and low-aggregation regions is also obvious. When comparing the results of the spatial autocorrelation on the district and street levels, except for some streets in Luojiashan and Xujiapeng, Shouyi Road, and Nanhu, the distribution of “High-High” population aggregation areas obtained by the two methods is very similar. The “Low-Low” population clustering characteristics on the street scale are more obvious than those on the district scale.

As shown in Table 5, more than half of the regions in Wuchang District have no population. Most grid cells have less than 25 people, while the grid cells with more than 300 people account for only approximately 3% of the total grid cells. The number of grid cells shows a significant decrease as the number of people increases, indicating that living space in Wuchang District is relatively decentralized and that the number of population-concentrated areas is relatively small. 

When compared with the use of the method on the district level, which shows a strong correlation between population results and these features, the use of the method on the street level can better reflect the geospatial uncertainty. Of the examined features, medical and health resources are the most sensitive to population concentration. Furthermore, as compared with the other features, these services are associated with a higher percentage of the population in the nearest buffers. The government has no apparent sensitivity to the extent of population aggregation. Distance increases as the number of people increasing, and the trend is rapidly decreasing in areas with a population of more than 1200. Educational resources and road networks have a relatively small impact on the population and cover nearly 80% of the population in the first buffer area. As shown by the overlay, most of the areas with large populations are close to governmental locations with medical and health and educational resources and well-developed road networks. Although road networks are not well developed in some parts of Luojiashan and Shuiguohu, there are universities and abundant educational and medical care resources nearby, so the population is large too. The Baishazhou and Nanhu streets are far from the urban center. These areas have poor road networks, and the population is small.

### 4.3. The Results of Cross-Validation Analysis

**Figure 11:**
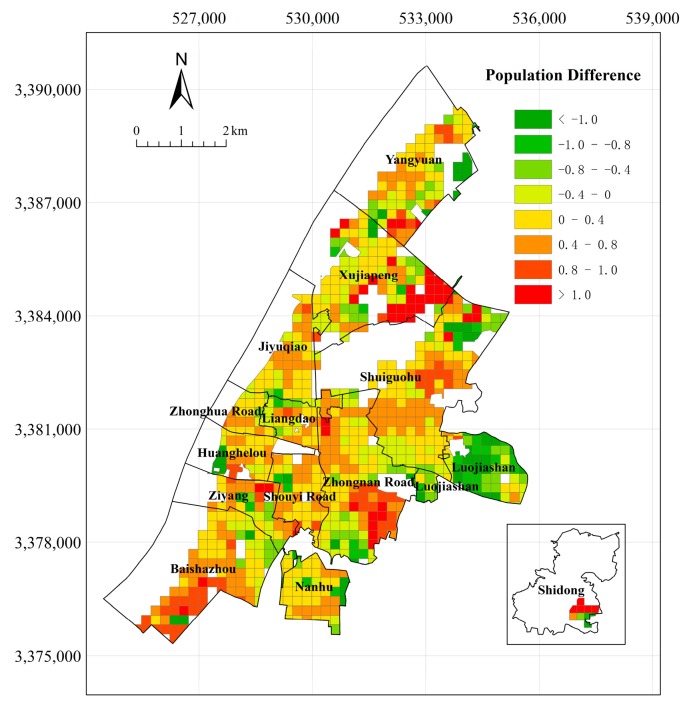
Population Difference According to Cross-validation Analysis.

The average deviations of the population that were covered by government, educational resources, medical and health resources, and road networks in buffer zones were 7.98%, 0.91%, 3.68%, and 7.56%, respectively, and the correlation coefficient of the results that were obtained by the two methods was 0.59. This analysis shows that the population within the coverage of the impact factor is relatively small, and that the results of the two methods are highly consistent. A thematic map in the population in Wuchang District on the different levels is shown in Figure 11.

Data on differences in the population results are collected, and the results are shown in Table 6.

Table 6 shows that the population results obtained from the two levels are not significantly different.61.84% of the population difference value is between −0.4 and 0.4, and only approximately 9% of the results are less than −1 or greater than 1. Large population differences are concentrated in the marginal areas of Wuchang District, such as Xujiapeng, Zhongnan Road, Baishazhou, Shidong, and Luojiashan. When combined with the areas of residential houses, the type of residential houses, and the residents of the community, these communities that are far from the center of Wuchang District have a small number of people and large areas of residential houses. Therefore, the estimated population on the district level is larger than that on the street level. Regarding Luojiashan, the distributions of the population and the residential houses are much more concentrated than those of the other streets because of the large terrain undulations. Additionally, several communities have many residents and small areas of houses, so the estimated population on the street level is greater than that on the district level.

The following conclusions can be drawn that are based on the above analysis: the population spatialization method on the district level can better highlight the spatial distribution of the population from the macro perspective. This method focuses on the impact of different types of residential houses on population density. This method is suitable when house areas are sufficiently large and the distribution of population and types of houses are diversified. Meanwhile, the population spatialization method on the street level can better display the present situation of the spatial distribution of the population from the micro perspective. This method focuses on calculating the impact of the residential houses within a small region on the population of the community. It is suitable for areas where the type of residential houses is simple and the house areas are small.

### 4.4. The Evaluation of Population Fit Accuracy

This paper uses the 1-km population grid data set of China from the National Earth System Science Data Sharing Infrastructure (http://www.geodata.cn), which established multivariate statistical models for populations in 1-km pixels in 2010 in China based on the correlations between the population and land use types. Urban population density, traffic conditions, DEM, and so on were used for model correction and forty counties with township population data from eastern, western, and central of China were chosen for precision verification.

**Figure 12:**
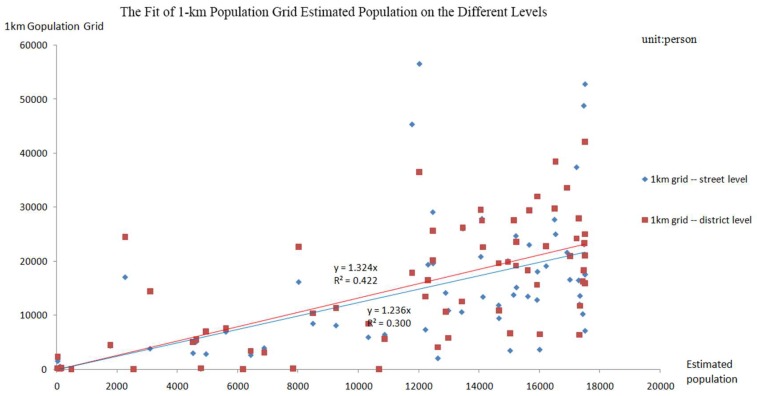
The Correlation between the Grid Population Data set and the Estimated Population.

To solve the problem of grid size mismatch between the two results, this paper spatially aggregates the population results on the street level and the district level, and the size of the merged grid is consistent with the 1-km population grid data set. The number of effective population grids has been adjusted to 66 by excluding the population grid around Wuchang District and avoiding a large population error due to the lack of data on housing construction in other urban areas. The fit analysis was performed on the estimated population on the district level and on the street level. The obtained results are shown in Figure 12. 

As shown in Figure 12, the goodness of fit on the street level is slightly lower than that on the district level, and the fitting coefficient on the street level is closer to 1 than that on the district level. The results on the district level have higher accuracy, but the results on the street level have a lower coefficient sensitivity. Thus, the results of the two levels are highly accurate and they have their own advantages.

## 5. Conclusions

This paper fully mines the Geographical National Monitoring Data to establish a multi-level population spatialization method for the different administrative levels. It also uses spatial analysis methods to explore correlations and influencing features, and finally verifies the accuracy of the fit results.

(1) The average fitting error in Wuchang District is only 13.03%, the fitting coefficient reaches 0.936, and the overall population estimation accuracy reaches 99.98% after building reclassification. The overall population estimation accuracy is 99.97% on the street level. The results truly reflect the spatial distribution of the population on the different levels.
(2) The spatial correlation in most areas of Wuchang District is not obvious through the spatial autocorrelation analysis results. In regions where the correlation is obvious, there is a large proportion of areas in the “High-High” condition on both levels. The distributions of the “High-High” population aggregation areas that were obtained by the two methods are highly similar, and the “Low-Low” areas on the street level are more obvious.
(3) In most areas, geographical location and road network are the dominant features that promote population aggregation. In other areas, the availability of public service can attract population aggregation, despite less convenience in terms of traffic.
(4) The population results that were obtained from the two levels are not significantly different; more than 60% of the population difference value is between −0.4 and 0.4. The average deviations of the population covered by different factors in the buffer zones were 7.98%, 0.91%, 3.68%, and 7.56%, and the correlation coefficient of the results obtained by the two methods was 0.59.
(5) When comparing the accuracy of experimental results against the 1-km population grid data, the fitting coefficient is 1.324, and the goodness of fit is 0.422 on the district level, while the fitting coefficient is 1.236 and the goodness of fit is 0.300 on the street level. The results of the two levels are highly accurate and have their own advantages.

However, the experiment found some deviations from relying solely on China’s first national geoinformation survey. The extraction of building construction may consider non-residential buildings or non-demolished houses, which may impact the calculation of population density. Future research will consider taxi trajectory data to solve this problem. The population density on the street or community level can be divided through the mining of taxi trajectories and analysis of residents’ activity patterns and activity areas. Then, differences in the spatial distribute of residents can be explored and the impact of different types of residential houses and regional locations on population activities can be analyzed. Finally, a model is established to estimate the population distribution status in residential areas at a smaller scale, and to improve the accuracy of population spatialization.

## Figures and Tables

**Table 1:** Detailed data usage list.

Data Name	Data Sources	Data Interpretation	Data Format
House building	The LCA layer in the Land Cover Classification Data	House buildings refer to the urban and rural areas of residential areas of housing construction, according to the attributes can be divided into 5 types	Shapefile
Urban integrated functional Units (point/area)	The BUCP and BUCA layers in Social Geographical Units	The Space unit divided by function and ownership within the urban residential areas. Including residential quarters and non-residential quarters (industrial and mining enterprises, institutions and companies). The difference between BUCA layer and BUCP layer is that BUCA layer contains surface vector features while BUCP contains point vector elements	Shapefile
District and street level administrative divisions	The BOUA5 and BOUA6 layers in Social Geographical Units	Vector data with region and district and street boundary	Shapefile
Community resident population and Community level administrative division	Wuhan Community Demographic Census	Community vector boundary with community resident population	Shapefile
Street resident population	The Sixth National Population Census	All streets resident population data in Wuhan	Excel
District resident population	Wuhan Statistical Yearbook	All counties resident population in Wuhan	Excel

**Table 2:** Detailed Description of House Buildings.

multi-floor buildings	More than 10 m in height or over four floors, construction area is more than 1600 m^2^. Mostly in densely populated areas in the central city
multi-floor independent buildings	More than 10 m in height or over four floors, construction area is more than 200 m^2^. Most buildings are scattered with low population density
low-floor buildings	Less than 10 m in height or four floors below, construction area is more than 1600 m^2^. Mostly high-grade residential quarters or planning township gathering area
low-floor independent buildings	Less than 10 m in height or four floors below, construction area is more than 200 m^2^. Most buildings are in rural areas where the economy is lagging behind and there are no plans for housing construction.
abandoned house building	Abandoned buildings after the migration

**Table 3:** Statistics of Corrected Coefficient Divided by Street.

Name	Street Population (P)	Multi-Floor + Multi-Floor Independent	Low-Floor + Low-Floor Independent	Estimates Population (P)	Fitting Error (%)	Coefficient 1	Coefficient 2
Baishazhou	78,676	535,235	792,363.4	99,206.82	−26.10	0.087527	0.040169
Huanglelou	52,713	282,799	307,857.4	46,805.24	11.21	0.124299	0.057044
Jiyuqiao	61,329	495,492.2	142,078.4	61,882.9	−0.90	0.10938	0.050198
Liangdao	64,008	393,565.1	458,598.3	66,665.45	−4.15	0.105968	0.048632
Luojiashan	62,574	564,579.4	228,263.9	73,873.29	−18.05	0.093487	0.042904
Nanhu	53,159	839,957.9	91,927.3	97,360.69	−83.14	0.060261	0.027655
Shidong	4618	11,322.39	74,066.45	5001.169	−8.30	0.101912	0.04677
Shouyi Road	69,872	511,765	225,943.7	67,926.76	2.78	0.113529	0.052102
Shuiguohu	172,007	1,397,723	263,553.6	167,613.1	2.55	0.113261	0.051979
Xujiapeng	122,129	1,171,513	557,920.3	157,556.8	−29.01	0.085551	0.039262
Yangyuan	109,245	840,912.3	357,030.3	110,893.7	−1.51	0.108727	0.049898
Zhonghua Road	44,693	276,456.8	370,415.9	49,273.92	−10.25	0.100107	0.045942
Zhongnan Road	234,479	1,216,299	550,660.3	162,132	30.85	0.159617	0.073253
Ziyang	52,770	253,579.2	242,408.3	40,265.25	23.70	0.144644	0.066381

**Table 4:** Population Coverage by Multiple Buffers.

Coverage Degree (%)	District Level				Street Level			
	Government	Educational Resources	Medical and Health Resources	Road Network	Government	Educational Resources	Medical and Health Resources	Road Network
Seed	2.17	20.66	13.28	--	2.07	20.11	11.68	--
First (50 m) buffer	16.19	82.40	63.99	92.67	15.50	82.96	62.82	76.84
Second (100 m) buffer	34.21	96.81	87.70	95.32	32.00	95.95	85.35	90.66
Third (150 m) buffer	51.69	99.22	95.12	96.97	46.15	99.21	93.68	92.63
Forth (200 m) buffer	67.15	99.70	97.72	97.68	57.85	100	97.42	93.96

**Table 5:** Statistics of the 50-m Grid Population is obtained through counting the population spatialization results on the street level.

Population	Count (N)	Proportion (%)
0	15,711	54.94
(0,25]	3530	12.34
(25,50]	2342	8.19
(50,100]	3192	11.16
(100,150]	1637	5.72
(150,200]	744	2.60
(200,300]	745	2.60
(300,450]	441	1.54
(450,700]	140	0.49
(700,1200]	104	0.36
>1200	13	0.06
Total	28,599	100

**Table 6:** Statistics of Population Differences on the Different Levels.

Difference	Count (P)	Proportion (%)
<−1.0	62	4.77
[−1.0–−0.8)	33	2.54
[−0.8–−0.4)	73	5.61
[−0.4–0)	133	10.23
0	418	32.15
(0–0.4]	253	19.46
(0.4–0.8]	209	16.08
(0.8–1.0]	68	5.23
>1.0	51	3.93
Total	1300	1
